# Virtual Reality to Improve the Psychological and Physical Well-Being in Cancer Patients: An Umbrella Review

**DOI:** 10.3390/cancers16233943

**Published:** 2024-11-25

**Authors:** Lucie Bachelard, Aude Michel, Nathalie Blanc

**Affiliations:** Epsylon Laboratory EA 4556, Paul Valéry University Montpellier 3, 34090 Montpellier, France; lucie.bachelard@etu.univ-montp3.fr (L.B.); aude.michel@univ-montp3.fr (A.M.)

**Keywords:** cancer, virtual reality, well-being, umbrella review

## Abstract

Background/Objectives: Cancer patients often experience psychological and physical symptoms that can significantly impact their quality of life and treatment outcomes. Virtual reality (VR) has emerged as a promising complementary approach to standard cancer care. Due to the rapidly evolving nature of VR research in oncology, it seems essential to provide an overview of the current state of knowledge in this field. Therefore, this umbrella review aimed to synthesize evidence from all relevant systematic reviews and meta-analyses on VR-based interventions for improving psychological and physical well-being among cancer patients. Methods: A comprehensive search was conducted in four databases (Cochrane Database of Systematic Reviews, Medline, Web of Science, PsycInfo) from inception to August 2023. We included systematic reviews with or without meta-analyses that evaluated the effects of VR-based interventions on any health-related outcomes in cancer patients. The methodological quality of included reviews was assessed using the AMSTAR-2 (A MeaSurement Tool to Assess systematic Reviews) tool. Results: Eighteen individual systematic reviews including ten performed meta-analyses were included, with sample sizes ranging from 225 to 842 participants. VR-based interventions were categorized into three main types: distraction, rehabilitation, and education. The interventions varied significantly in terms of content, equipment, setting, and duration. The most frequent use of VR is one-off exposure in hospital settings to distract patients from anticancer treatment and painful procedures, with natural or game-based content. The mechanism most commonly proposed to explain the effectiveness of VR involves attentional redirection. Conclusions: This umbrella review suggests that VR-based interventions may be effective in improving psychological outcomes in cancer patients, particularly anxiety, pain, and depression. However, high heterogeneity across studies and mixed results for some outcomes indicate a need for further high-quality research. Moreover, a more refined analysis of VR-based interventions is essential to explore the specific mechanisms of action associated with each type. This approach would enable us to establish the optimal characteristics of each intervention and their long-term effects.

## Simple Summary

The use of virtual reality to support cancer patients has gained increasing interest in recent years, leading to a rise in systematic reviews and meta-analyses. However, no umbrella review has yet been carried out to synthesize the current state of evidence. The aim of this umbrella review is to fill this gap, providing a comprehensive overview of the various virtual reality-based interventions in oncology, and examining their effects and limitations. This synthesis provides a framework to guide future research and enable the scientific community to direct the development and optimization of virtual reality technologies to improve the cancer care pathway.

## 1. Introduction

Cancer is a common pathology that represents a major challenge for public health worldwide. Each year, an increasing number of new cases are diagnosed, reflecting a continually rising incidence [1]. Patients diagnosed with cancer must cope with various symptoms such as anxiety [2], depression [2], pain [3], fatigue [4], and cognitive impairments [5]. Lack of psychological management of these symptoms can lead to impaired quality of life [6,7] and cancer prognosis (increased cancer-specific mortality and decreased cancer survival) [8]. In this context, the need to develop complementary approaches to standard treatments is well established.

Given the challenges associated with the dynamics of hospitalization and time constraints, virtual reality (VR) appeared to be a promising technology for supporting cancer patients [9]. VR enables a user to interact in real time with a computer-generated 3D virtual environment. VR typically involves the use of a headset for complete immersion (i.e., retransmission of an image covering the entire field of vision) [10]. However, other devices can also be used, such as nonimmersive systems (e.g., computer screen), which offer more limited immersion, and semi-immersive systems (e.g., Cave Automatic Virtual Environment), which provide partial immersion in a dedicated physical space [10].

Numerous studies have explored the use of VR at various stages of the care pathway, highlighting its diverse potential applications and effects on psychological, physical, and biological variables [11]. VR has been shown to be beneficial for emotional management during chemotherapy infusions and hospitalizations [11], as well as for its analgesic effects during painful procedures [11], notably by immersing patients in distracting and relaxing virtual environments. VR-based interventions can also take the form of personalized integrative rehabilitation games, improving certain physical and cognitive functions after oncological treatment [12]. More recently, VR emerged as a promising solution for delivering medical information, improving understanding of complex treatments (e.g., radiotherapy, surgery) [13], and reducing treatment-specific anxiety [14]. In addition to the benefits for patients, VR has a significant potential for healthcare systems by possibly reducing medication requirements [15], shortening hospital stays [16], and improving treatment adherence [17], which could lead to substantial cost savings and improved resource allocation.

The growing number of clinical trials evaluating the effects of VR on patients’ mental and physical health has been accompanied by a similar rise in the number of reviews. However, to the best of our knowledge, no umbrella review has been conducted to synthesize evidence from systematic reviews and meta-analyses on VR-based interventions in cancer patients. Our review aims to fill this gap by providing a comprehensive analysis that includes patients of all cancer types and age groups, acknowledging that cancer affects individuals throughout their lifespan. This inclusive approach enables us to provide a narrative analysis of the different fields of application of VR in oncology and the characteristics and theoretical aspects underlying these interventions. By doing so, we aim to better understand the mechanisms of action of VR, identify existing gaps in the available evidence, and explore VR applications across the entire spectrum of cancer care. This comprehensive overview will help accelerate the updating of knowledge and guide clinical practice and future research in this rapidly evolving field.

## 2. Materials and Methods

This umbrella review is presented according to the guidelines of the Preferred Reporting Items for Systematic Review and Meta-Analysis (PRISMA) statement [18]. The completed PRISMA checklist is available in the Supplementary Files. In line with these recommendations, our protocol is registered with the International Registry of Prospective Systematic Reviews (PROSPERO) (CRD42023469769). No ethical approval is required for this review as we rely exclusively on previously published studies in the literature.

### 2.1. Eligibility Criteria

We followed the PICOS checklist described by PRISMA [18] for eligibility criteria: (1) types of population, all types of cancer patients (children and/or adults); (2) types of intervention, any VR-based interventions; (3) types of comparator, compares the intervention to any group or control group; (4) types of outcome, any psychological (e.g., anxiety, pain), cognitive (e.g., knowledge), physical (e.g., muscle, range of motion), or biological (e.g., heart rate, cortisol levels) variable; (5) types of study, systematic reviews (with or without meta-analysis). Studies were excluded if (1) they were not published in English or French, (2) if the full text was not available, and (3) if they described only a protocol.

### 2.2. Information Sources and Search Strategy

Two reviewers (L.B. and A.M.) independently searched the following databases from their creation to August 2023: Cochrane Database of Systematic Reviews, Medline (PubMed), Web of Science, and PsycInfo. The following terms were searched in the title, abstract, and keywords of the publication: (“virtual reality” OR “immersiv* technolog*” OR “virtual environment” OR “virtual reality-based intervention”) AND (“cancer” OR “oncolog *” OR “malignan *” OR “tumor” OR “neoplasm”) AND (“systematic review” OR “meta-analys *” OR “scoping review”). The search method was adapted to suit the different databases consulted. Once the search was complete, the references were imported into Excel (Microsoft Office, version 2016), and duplicates were eliminated.

### 2.3. Selection Process

Two independent reviewers (L.B. and A.M.) used the above eligibility criteria to examine titles and abstracts in order to identify potentially relevant journals. After consultation, if a decision could not be made based on the title and abstract alone, the full review was retrieved. The full texts of the remaining studies were reviewed independently by two reviewers (L.B. and A.M.). Any disagreements were resolved by a third reviewer (N.B.).

### 2.4. Data Extraction Process

Two independent reviewers (L.B. and A.M.) extracted data from all reviews using a predefined data extraction form. Any disagreements were discussed with a third reviewer (N.B.). The data extracted included the following:

- Review identification data (i.e., authors, year of publication, country of origin)
- Review characteristics (i.e., whether a meta-analysis was performed or not, objective(s), number and types of studies included, number of participants)
- Population characteristics (i.e., age group, diagnosis)
- Characteristics of intervention and control groups (i.e., intervention contents, equipment, settings, length and frequency, underlying theoretical aspects)
- Main conclusions: statistical significance reported in the reviews, including effect measures such as mean difference, standardized mean difference, and I-squared values, was examined to evaluate the authors’ conclusions.

### 2.5. Risk of Bias Assessment

Two reviewers (L.B. and A.M.) independently assessed the quality of all included reviews. Differing opinions were discussed with a third reviewer (N.B.) until a consensus was reached. The measurement tool AMSTAR-2 (A MeaSurement Tool to Assess systematic Reviews) [19], which consists of a checklist of 16 items, was used to assess the methodological quality of the reviews. It is worth noting that AMSTAR-2 is not designed to provide an overall score [19]. It highlights the importance of flaws in critical areas that can significantly impact confidence in the conclusions of systematic reviews. The Supplementary Files provides a detailed description of all items.

## 3. Results

**Figure 1:**
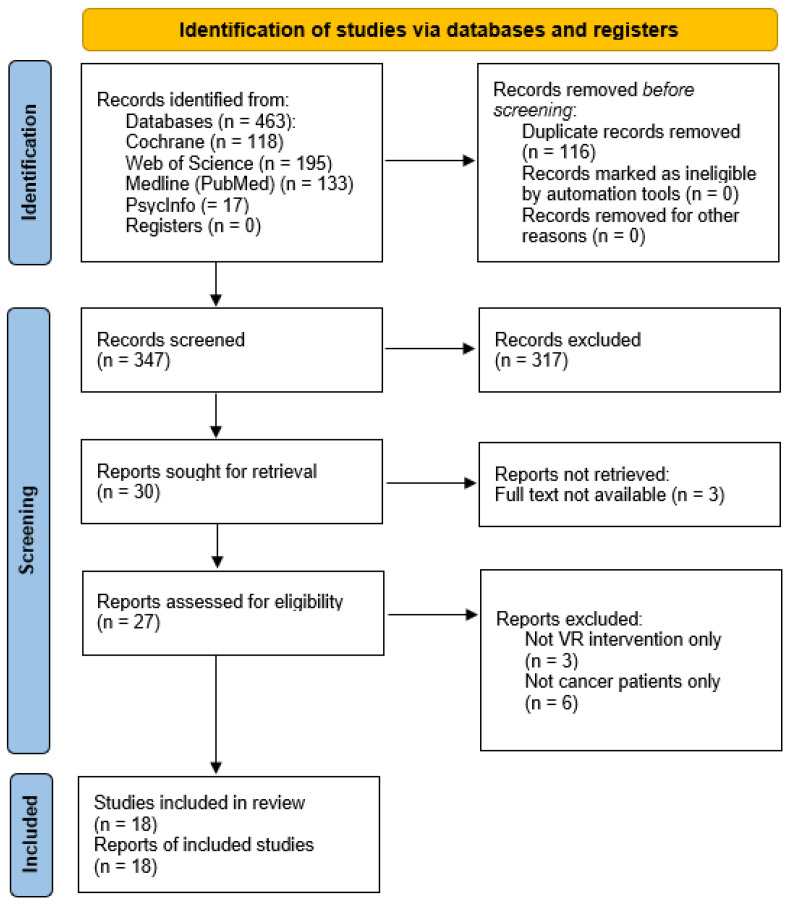
PRISMA flow diagram of the review selection.

The electronic search in the four databases generated a total of 463 records. Of these, 116 were removed before screening because they were duplicate records. After removing duplicates, the titles and abstracts of the remaining 347 articles were screened. Three hundred and seventeen articles were excluded because they did not meet the inclusion criteria. The main reasons for exclusion were that they focused on other types of interventions and/or populations, or the full text was not available. Finally, 18 reviews were selected for this umbrella review. The full search results are illustrated using the PRISMA flow diagram (Figure 1).

### 3.1. Characteristics of Included Reviews

All included reviews were published between 2019 and 2023 in countries such as China (6/18), Italy (3/18), Poland (3/18), Australia (1/18), the United States (1/18), Portugal (1/18), Iran (1/18), Taiwan (1/18), and Jordan (1/18). The reviews synthesized findings from between six and twenty studies. Sample sizes ranged from 225 to 842 participants; two reviews did not provide details about sample sizes [20,21]. After removing duplicate entries from the systematic reviews, we identified a total of 71 primary studies. Ten of the eighteen included reviews also performed a meta-analysis on randomized controlled trial (RCT) results (3/18), or combined RCT results with quasi-experimental design results (7/10). The most commonly used type of control group is standard care (e.g., painkillers only, pretreatment teaching, standardized physical therapy, conscious sedation). Some reviews included visualization on a two-dimensional screen as a comparator (4/18), music therapy (4/18), or other types of integrative therapies (e.g., guided imagery, green therapy) (1/18). Two reviews did not report any information about the control group [22,23]. The main clinical outcomes of interest were anxiety (18/18), pain (15/18), depression (9/18), and fatigue (9/18), followed by physical (5/18) and cognitive (4/18) function outcomes. The majority of studies assessed symptom outcomes using self-administered questionnaires. Most of the included reviews provide an overall perspective on VR in oncology, regardless of population age or diagnosis. However, some have focused exclusively on adult (9/18) or pediatric patients (3/18), breast cancer patients (5/18), patients with hematological or solid cancers (1/18), and patients receiving chemotherapy (3/18) or radiotherapy (1/18) (Table 1).

### 3.2. Risk of Bias of the Included Reviews

The assessment of the included systematic reviews with the AMSTAR-2 checklist revealed significant gaps in the critical domains (Table 2). Five reviews (27%) developed an a priori protocol (item 2). Although all reviews conducted a comprehensive literature search (item 4), none could be rated as “Yes” due to the failure to meet the requirement of consulting content experts in the field (item 4). Six reviews (33%) provided a list of excluded studies and justified the exclusions (item 7). Only one review (5%) did not employ an appropriate method for evaluating the risk of bias (RoB) in individual studies (item 9). However, although the majority of studies correctly assessed the risk of bias (RoB), only five studies (27%) took these biases into account when interpreting and discussing the results (item 13). The majority of the meta-analyses conducted (80%) employed appropriate methods for the statistical combination of results (item 11). However, there was limited investigation into the sources of heterogeneity, primarily due to the insufficient number of individual studies included in the analyses. Furthermore, many meta-analyses failed to appropriately separate RCTs from Non-Randomized Studies of Intervention (NRSIs). Regarding the last critical domains related to meta-analysis, only two reviews (11%) performed an investigation of publication bias (item 15). It is worth noting that none of the reviews mentioned the funding sources of the included studies (item 10). Finally, an important concern emerged regarding item 8: only six reviews (37.5%) provided detailed information on the timeframe for follow-up. This indicates that, although interventions are generally well described, evaluation methods often lack essential details. It remains unclear whether this shortcoming stems from a lack of rigor in the reviews themselves or from insufficient information in the underlying primary studies. Regardless, this finding highlights the need for greater transparency and more rigorous documentation, particularly to ensure consistent and reliable evaluation of the studied interventions.

## 4. Report Conclusions and Summary of Evidence

Three main forms of VR interventions have been described in the current literature: VR-based distraction, VR-based rehabilitation, and VR-based education. Of the 18 reviews examined, 7 included only VR-based distraction interventions in their analysis [24,26,29,30,32,35,36] and 1 focused exclusively on VR-based education interventions [34]. The remaining reviews combined results from different types of interventions. The following section presents a narrative analysis of VR-based interventions in oncology. A summary of findings is reported in Table 3.

### 4.1. Characteristics of VR-Based Interventions

#### 4.1.1. Intervention Objectives

VR-based distraction interventions aim to provide temporary relief of symptoms associated with cancer and its treatments (e.g., anxiety, pain, depression, fear), by allowing patients to escape from the medical setting and immerse themselves in a pleasant environment [22]. VR-based rehabilitation interventions aim to improve patients’ cognitive and physical functions through exercises and simulations of everyday activities [25,33]. VR-based educational interventions aim to provide patients with information including detailed explanations of medical processes [20,34], in order to alleviate apprehension and enhance comprehension of complex treatments.

#### 4.1.2. Intervention Contents

A variety of content is used to support patients, ranging from relaxing natural scenes to interactive games and specific medical simulations. The virtual environments most commonly deployed are representations of natural landscapes, mainly derived from commercially available applications (e.g., Oceans Below, Ocean Rift, Happy Place). Of the sixteen studies reviewed, only one did not report the use of this type of content in its analysis [34]. Some interventions are based on simulations of everyday tasks, interactive games, or sports training (e.g., tennis, rhythmic boxing, beach volleyball) [21,22,24,25,27,31,33,35]. To a lesser extent, a few reviews have included interventions with specific programs incorporating information content related to treatment procedures [20,22,27,34].

**Table 3:** Intervention characteristics.

Author(s) (Year)	Type of Interventions Included	Intervention Contents	Intervention Settings	Intervention Equipment	Intervention Length and Frequency	Underlying Theoretical Aspects	Main Conclusions
Ahmad et al. (2020) [9]	NS	NS	During painful procedures (5 studies), anticancer treatment (6 studies), or hospitalization (2 studies)	Immersive (glasses and HMD) and nonimmersive (1 study only, NS)	Sessions lasted from 3 to 5 min during painful procedures, from 45 to 90 min during chemotherapy and fusions, and about 30 min during hospitalization; there was only one session	None	VR is an effective adjuvant intervention, particularly for reducing pain and anxiety in children and adolescents during painful procedures, as well as for reducing anxiety in adults and the elderly during chemotherapy, RT, or hospitalization. However, the differences are not always statistically significant.
Bu et al. (2022) [25]	VR-based distraction, VR-based rehabilitation, VR-based education	Interacting with nature-related virtual environments (e.g., relaxing landscapes, Oceans Below), following a VR program including RT-related content, VR exercises and training	Before RT (1 study), during chemotherapy (3 studies) or hospitalization (1 study), after surgery (6 studies) or chemotherapy (1 study)	Immersive (HMD), semi-immersive (VERT), and nonimmersive (Xbox Kinect, Nintendo Wii, The BrightArm Duo Rehabilitation System, VR-based Rehabilitation System); other NS	Sessions lasted from 15 min to 90 min (M = 36 min), with a frequency ranging from 1 single session to 2 per day, over a period ranging from 4 weeks to 12 weeks	Refers to the attentional and distraction mechanism	Meta-analyses were performed on shoulder ROM, hand grip strength, anxiety, depression, pain, and cognitive function, showing significant positive results in favor of VR-based interventions over standard care (except for hand grip strength). However, there was substantial heterogeneity in most cases. The authors highlight the need for studies with a more robust design and sample size. There is also a need for standardization of intervention content (to properly implement VR in daily practice, more information is needed on the frequency of training, the duration of the intervention, and the motor skills targeted).
Burrai et al. (2023) [29]	VR-based distraction	Mostly interacting with nature-based virtual environments (e.g., relaxing landscapes, lakes, mountains, tropical beaches)	During chemotherapy only	Immersive (HMD) and nonimmersive (three-screen LCD)	Sessions lasted from 5 min to 20 min or could last as long as the patient wished; there was only one session	Attentional mechanism: shift attention from unpleasant current experiences towards pleasant, interesting stimuli, by re-shaping the perceived environment	Meta-analyses revealed no significant difference in fatigue and pain, either between VR and the control group or VR and integrative therapy. However, a significant improvement in anxiety appeared in favor of VR compared to the control group (with substantial heterogeneity). It should be noted that when compared with integrative therapy, VR did not provide significantly better results. Once again, the methodological quality of the included studies is criticized: none of the studies included after 2010 followed CONSORT standards.
Cheng et al. (2022) [26]	VR-based distraction	Virtual simulation experiences, game-based (e.g., rollercoaster) or nature-based (e.g., watching in the eyes of an animal, Australian national parks or zoos, ocean journey), playing a game, watching VR cartoons	During painful procedures (4 studies), chemotherapy (1 study), or hospitalization (1 study)	Immersive only (HMD)	Before and during the procedure; no more details	Attentional mechanism: stimulation of multiple senses to reduce the perception of pain and anxiety, redirecting neural signals and shifting harmful stimuli to neutral or pleasant ones	Significant positive effects of VR on all variables measured (i.e., pain, anxiety, and fear). High heterogeneity exists in terms of instruments used, type of cancer, and control group. The included studies used various types of VR (e.g., interactive games, videos, more or less interactive simulations) and scenarios, making it difficult to determine which scene may be most effective in reducing pain and anxiety.
Chow et al. (2021) [24]	VR-based distraction	Interacting with virtual environments (e.g., Snow World, Titanic: Adventure Out Of Time, Oceans Below), watching videos of relaxing nature scenes or videos showing various experiences (e.g., skiing, racing), exploring a gorilla habitat in the zoo	During painful procedures (5 studies) or chemotherapy (4 studies)	Immersive only (HMD)	Sessions lasted from 3 min to 78 min (M = 57 min); there was only one session	Attentional mechanism: the brain has a limited capacity to process multiple sensory inputs from various stimuli. In this regard, immersion in a virtual environment may divert attention from pain and/or anxiety, which could explain the therapeutic effect of VR	Quantitative data showed no significant improvement in anxiety, and only two out of five studies evaluating pain reached statistical significance. Interestingly, these two studies are the only ones that used active VR. Future research should be more rigorous.
Comparcini et al. (2023) [30]	VR-based distraction	VR scenarios, game-based (e.g., Sherlock Holmes Mystery, Roller Coaster video, Rilix VR) or nature-based (e.g., Australian natural parks, ocean journey), animated videos	During oncological procedures (8 studies), active treatment or hospitalization	Immersive (HMD), semi-immersive (video projector), and nonimmersive (computer screen)	NS, but usually for the duration of the procedure	Refers to the distraction mechanism	There is a beneficial effect of immersive VR on pain. Regarding nonimmersive VR, no significant effect on pain was observed. There is insufficient and inconsistent evidence despite the beneficial effect of immersive VR on anxiety.
Czech et al. (2023) [31]	VR-based distraction	Animated videos, nature-based content, rollercoasters, games	During chemotherapy (1 study) or oncological procedures (4 studies), after cancer treatment (3 studies); other NS	Immersive (HMD) and nonimmersive (Nintendo Wii, Xbox Kinect)	Sessions lasted from a few minutes during the procedure to 45 min per day, with a frequency ranging from 1 session to 5 sessions per week, over a period ranging from 8 weeks to 12 weeks	Refers to the attentional and distraction mechanism: reducing the sensory and affective components of pain and the ability to distract attention	Meta-analyses revealed significant differences in all outcomes (i.e., anxiety, pain, and fear) but the results were too heterogeneous to be pooled.
Gautama et al. (2023) [35]	VR-based distraction	Adult: Exploring natural landscapes (e.g., sea, lakes, forests, beaches, waterfalls, mountains), walking through an art museum, watching a concert, yoga sessions. Pediatric: VR scenarios, game-based, outdoor activities, cartoon movies	Chemotherapy (before, during, and after)	Immersive only (HMD)	Sessions lasted from 5 to 90 min; there was only one session	Refers to the attentional and distraction mechanisms	Adult patients showed a significant decrease in anxiety, depression, and fatigue immediately after VR, compared to the control group. However, no significant difference emerged in distress and pain. Similarly, no significant differences were revealed after 48 h of immersion in anxiety, depression, and fatigue. Pediatric patients showed a significant decrease in anxiety and pain compared to the control group. There were inconclusive results regarding physiological indicators, regardless of age group.
Grilo et al. (2023) [34]	VR-based education	Sessions attended individually (4 studies), in a group (1 study), or in both modalities (3 studies); VR videos or VERT to present the various stages of the procedure	Before the CT scan (3 studies) or treatment (4 studies) or on the first day of treatment (1 study)	Immersive (HMD) and semi-immersive (VERT)	Sessions lasted from 30 min to 1 h; there was only one session	Refers to the attentional mechanism: modulating emotional processes and reducing pain-related brain activity and anxiety. In its informative role, VR helps meet patients’ information needs (increasing knowledge and ultimately reducing anxiety)	Pre-RT educational sessions appear to be effective in improving patients’ understanding of the procedure. Results are less homogeneous concerning anxiety levels. The included studies have low methodological quality (many questionnaires were constructed for the specific needs of the studies) and high heterogeneity. Further trials are needed to establish the beneficial effect of VR as an educational intervention.
Leggiero et al. (2020) [36]	VR-based distraction	Mostly nature-based virtual environments (e.g., scenarios related to ocean exploration, walking through forests and cities); no more details	During chemotherapy (3 studies), hospitalization (5 studies), or stressful medical procedures (2 studies); other NS	Immersive (HMD) and nonimmersive (television, computer screen)	Sessions lasted from 3 min to 2 h (M = 31 min), with a frequency ranging from 1 single session to 5 per week	None	There was an overall improvement in anxiety symptoms, but it was not statistically significant in all included studies. Results for other variables were mixed and inconclusive. There was high heterogeneity in the included studies (population, instruments used, VR approaches and duration, etc.). Studies with a more robust design and sample size are needed.
Rutkowski et al. (2021) [32]	VR-based distraction	Interacting with nature-related virtual environments (e.g., lake, forest, sea) and more scenic virtual environments such as puzzle solving (i.e., Titanic: Adventure Out Of Time)	During chemotherapy only	Immersive only (HMD)	Sessions lasted from 15 min to 90 min (M = 38 min); there was only one session	Refers to the distraction mechanism	The meta-analysis showed no significant differences in anxiety levels between the groups. Regarding fatigue, the meta-analysis could not be performed due to a lack of data. The poor quality of the publications does not provide conclusive results (e.g., small sample sizes, differences in study design).
Sansoni et al. (2022) [20]	VR-based distraction, VR-based education	Nature-based virtual environments or 360 videos (e.g., relaxing landscapes, Australian zoos, memorable places), animated videos, games, commercial VR scenarios (e.g., Magic Carpet, Sherlock Holmes Mystery), 360° videos simulating the RT or surgery experience, VR program including RT-related content	Before treatment (3 studies) or during active treatment (10 studies), during oncological procedures (5 studies) and palliative care (2 studies)	Immersive only (HMD)	Sessions lasted from 4 min to 90 min (M = 22 min); there was a single session for all studies except one	Lazarus and Folkman’s stress and coping model: faced with a stressful situation that they cannot change, people tend to adopt emotion-focused coping strategies (e.g., distraction) to regulate their emotions. In this sense, VR distraction is an emotion-focused method. Attentional mechanism: VR distraction works because of our mind’s inability to handle too much information at once (limited cognitive resources). On the other hand, educational studies employ the cognitive–behavioral strategy known as exposure. By exposing patients to simulations of their future medical experience, this reduces uncertainty and enables them to acquire knowledge about the different stages of the procedure. This exposure helps them to restructure their expectations, thus reducing anxiety before the start of treatment. The authors have defined this as “informative exposure”	The results reported are mixed but still point to the effectiveness of VR. The authors emphasize that VR-based education helps patients actively acquire skills to cope with their diagnosis, which is not the case with psychological interventions. Thus, they suggest that psychological interventions should enable patients to play a more active role.
Tian et al. (2023) [27]	VR-based distraction, VR-based rehabilitation, VR-based education	Interacting with nature-based environments (e.g., Ocean Rift, Happy Place, Oceans Below), VR exercises and training, VERT	During chemotherapy (1 study); other NS	Immersive only (HMD)	Sessions lasted from 10 to 60 min, from 1 to 180 sessions (twice a day for 12 weeks)	Refers to the attentional mechanism	Immersive VR was effective for anxiety, depression, and pain management in breast cancer patients.
Wu et al. (2023) [22]	VR-based distraction, VR-based rehabilitation, VR-based education	VR scenarios (e.g., exploring forests, zoos, playgrounds, museums, seasides), VR exercises, and simulation of the RT process	Before RT (1 study) or surgery (1 study), during oncological procedures (4 studies) or active treatment (1 study), home-based (1 study); other NS	Immersive (HMD) and nonimmersive (Xbox Kinect)	Sessions lasted from 10 min to 1 h, with a frequency from 1 single session to 5 sessions per week, over a period ranging from 6 weeks to 12 weeks	Refers to the distraction mechanism and exposure-based therapy (for VR-based education)	For all the variables evaluated except quality of life, the meta-analyses showed significant positive effects in favor of VR over control groups (with moderate to high heterogeneity).
Yazdipour et al. (2023) [23]	VR-based distraction, VR-based rehabilitation	VR exercises and relaxation techniques in VR; no more details	Before RT (1 study), during chemotherapy (7 studies) or oncological procedures (2 studies), after surgery (7 studies), in patients with a history of cancer (1 study)	Immersive (HMD) and nonimmersive (television screen, Xbox Kinect)	Sessions lasted from 7 min to 90 min, with a frequency ranging from 1 single session to 6 sessions per week, over a period ranging from 2 weeks to 8 weeks	Refers to the distraction mechanism	VR-based interventions showed positive effects on anxiety, pain, time perception, distress, fatigue, strength, and function metrics. The authors report on a number of challenges and limitations, especially related to VR (e.g., weight of headsets, user resistance because of first exposure to the VR, quality of visual image) and the studies included (e.g., small sample size, study design, risk of bias).
Zasadzka et al. (2021) [33]	VR-based distraction	Interacting with virtual environments (e.g., A World of Art, Titanic: Adventure of Time), mostly nature-based (e.g., Ocean Rift, Oceans Below, walking through a forest), VR exercises or games (e.g., boxing, table tennis)	After surgical removal of breast tumors (4 studies), during chemotherapy (6 studies) or hospitalization (1 study)	Immersive (HMD) and nonimmersive (Nintendo Wii, Xbox Kinect, The BrightArm Duo Rehabilitation System, Telko); Note: nonimmersive VR was used to improve motor functions of patients	Sessions lasted from 20 min to 90 min (M = 45 min), with a frequency ranging from 1 single session to 6 per week, over a period ranging from 3 weeks to 8 weeks	Refers to the redirection of attention	VR seems to have positive effects on certain aspects of the mental sphere (e.g., anxiety, fatigue, distress), pain, lymphedema, and some physical aspects (e.g., dynamic postural control, strength). However, it is difficult to draw conclusions given the lack of methodological quality and statistical power of the studies.
Zeng et al. (2019) [28]	VR-based distraction, VR-based rehabilitation	Interacting with virtual environments (e.g., urban park, forest, Ocean Rift, Happy Place), watching relaxing nature scenes, VR exercises	During hospitalization (3 studies) or chemotherapy (1 study), home-based (1 study); other NS	Immersive (goggles ezVision X4, HMD) and nonimmersive (television, Nintendo Wii Fit, VR-based Rehabilitation System)	Sessions lasted from 15 min to 50 min, with a frequency ranging from one-off exposure to five sessions per week, over a period ranging from 1 week to 16 weeks	None	Positive effects of VR were observed but only fatigue showed significant improvement. Significant heterogeneity exists among the included studies highlighting the need for conducting research with more robust study designs and larger sample sizes.
Zhang et al. (2022) [21]	VR-based distraction, VR-based education	VR exercises, visualizing the RT dose plan, interacting with nature-based environments (e.g., Ocean Rift, walking through a forest, mountain climbing)	After surgery (5 studies), during hospitalization (1 study) or chemotherapy (1 study), before RT (1 study)	Immersive (HMD), semi-immersive (VERT), and nonimmersive (Xbox Kinect, The BrightArm Duo Rehabilitation System, computer screen)	Sessions lasted from 15 min to 1 h (M = 30 min), with a frequency ranging from 1 single session to 6 per week, over a period ranging from 6 weeks to 12 weeks	Refers to the attentional mechanism: the immersion provided by VR shifts the patient’s focus of attention and offers a positive, pleasurable experience. It has also been shown that the interactivity of VR could activate the brain’s dopaminergic pathway, thus stimulating patient interest: the reward mechanism makes the intervention more attractive, encouraging patients to use VR	Meta-analyses were performed on anxiety, fatigue, and abduction only (too much heterogeneity between groups). The pooled effects showed that VR-based interventions had an effect on anxiety and abduction of upper limbs, but not fatigue. High heterogeneity exists between studies: in types of treatment, stages, and types of cancer, sample size, VR equipment, frequency, and duration of intervention (sometimes unclear).

Note: NS, not specified; HMD, head-mounted display; RT, radiotherapy; VERT, virtual environment for radiotherapy training; ROM, range of motion; M, mean; CT, computed tomography.

#### 4.1.3. Intervention Equipment

Immersive devices (i.e., head-mounted displays) are the most widely used equipment to provide the virtual experience. Six reviews [20,24,26,27,32,35] included only this type of device in their analysis. These devices allow for 360-degree immersion, completely encompassing the field of vision, and are therefore acknowledged as providing the highest level of immersion. Some interventions exploit gaming platforms such as the Xbox 360 Kinect or Nintendo Wii [21,22,23,25,28,31,33], as well as systems specifically designed for particular purposes, such as the BrightArm Duo [21,25,28,33] or the virtual environment for radiotherapy training (VERT) [21,25,34].

#### 4.1.4. Intervention Settings

These interventions are predominantly delivered in hospital settings during lengthy, anxiety-provoking, or painful oncological procedures (e.g., chemotherapy, bone marrow aspiration, venous port access), although some studies have explored their application in patients’ homes [22,28,31]. Additionally, VR-based interventions showed promise when implemented at the end of oncological treatment (e.g., after chemotherapy, during post-operative recovery) [21,22,25,31,33] or prior to complex treatments such as radiotherapy or surgical interventions [9,20,21,22,23,25,27,28,33,34]. They are mainly delivered individually, but some reviews included studies involving group sessions especially for educational sessions using VERT [34,36], although this was not always specified.

#### 4.1.5. Intervention Length and Frequency

The length of the interventions varies significantly from one study to another. The shortest reported length was 3 min [9,24,36] and the longest was 2 h [36]. The most frequent mean duration is about 10–15 min. VR-based interventions usually take the form of a one-off exposure [9,20,24,29,30,32,34], but the frequency varies according to the setting in which it is deployed. A single session in a clinical setting is common before and during treatment, while post-treatment interventions generally involve repeated sessions, for rehabilitation purposes. The highest frequency recorded is six times per week [21,23,33]. Some interventions were scheduled over a period of 16 weeks [28].

#### 4.1.6. Underlying Theoretical Aspects of VR-Based Interventions

Several theoretical mechanisms, mainly linked to the impact of VR on brain function, have been advanced to explain its potential therapeutic effect in medical settings. The attentional mechanism is the theoretical argument most widely evoked [20,21,23,24,25,26,27,29,31,32,34,35]. VR exploits the brain’s limited capacity to process multiple pieces of sensory information simultaneously. By immersing patients in a virtual environment, it diverts their attention away from painful or anxiety-provoking stimuli, thereby modulating their cognitive and emotional experience [24,26]. This cognitive distraction attenuates the perception of pain and anxiety. Multisensory VR stimulation could induce reorganizations in the cerebral cortex, which could have an impact on patients’ motor and cognitive functions [33]. In addition, immersion may act on certain areas of the brain, positively influencing motor integration functions (such as movement coordination) and spatial orientation (the perception of space and the ability to situate oneself in a virtual environment) [21]. Some authors suggested that VR-based interventions can enable patients to develop coping skills to deal with their disease. Based on Lazarus and Folkman’s [37] model of stress and coping, VR-based education can be viewed as a problem-solving method, allowing patients to increase their knowledge and adopt a more active role in their healthcare [20]. Conversely, VR-based distraction can be considered an emotion-centered method, as individuals tend to employ strategies such as distraction when they feel unable to change a stressful situation [20]. Moreover, VR-based education can be considered as an exposure-based technique, as it confronts patients with feared stimuli, thus restructuring their expectations and therefore reducing the associated situational anxiety [20,22].

### 4.2. Effects of VR-Based Interventions

#### 4.2.1. Psychological Outcomes

- Anxiety

All the meta-analyses included assessed the effect of VR-based interventions on anxiety [21,22,25,26,27,28,29,31,32,35]. Pooled data covered two [29] to seven studies [22]. Among them, only one did not find a significantly superior effect of VR compared to standard care [32]. Surprisingly, Burrai and colleagues [29] included the same preliminary studies as Rutkowski and colleagues [32] and found significant differences between groups. Heterogeneity was significant across all pooled anxiety outcomes, and two meta-analyses did not pool the data due to the level being too high [31,32]. However, subgroup analyses showed that when compared with an integrative intervention (e.g., guided imagery, music therapy), no significant effect emerged [29]. Additionally, the effects of VR on anxiety were no longer observed in the 48 h after the intervention [35]. Wu and colleagues [22] performed subgroup analyses on the type of VR content (landscapes vs. health education) and age group (juvenile vs. adult). The results indicated that VR was more effective than standard care in reducing anxiety symptoms, regardless of the analysis group. In all other reviews, the results are less homogenous [9,20,23,24,30,33,34,36]. The added benefit of VR was not systematically observed across the preliminary studies.

- Pain

Eight meta-analyses were conducted on pain outcomes [22,25,26,27,28,29,31,35]. Pooled data covered two [29,35] to seven studies [22]. Of these, six reported a significant effect in favor of VR over the comparison conditions. However, three analyses found no significant effect of VR compared to standard care [20,25,29] or integrative therapy (i.e., green therapy) [29]. Significant heterogeneity was reported across almost all pooled studies. Subgroup analyses conducted by Wu and colleagues [22] showed that VR-based interventions were significantly superior to control groups for improving pain, regardless of age group (juvenile vs. adult) or intervention content (landscapes vs. rehabilitation training). Regarding the findings reported by other reviews, VR appeared useful in addressing pain in most of the preliminary studies included [9,21,23,24,33,36]. However, Comparcini and colleagues [30] found no significant effect of immersive VR over comparison conditions (i.e., standard care and nonimmersive VR).

- Depression

Five meta-analyses covering two [22,25,27,28] to four studies [35] were carried out. Significant positive results favoring VR-based interventions over control groups were observed in all but one review [28]. Reported heterogeneity ranged from low [22,25,28] to high [27,35]. All the other reviews that analyzed this outcome reported a significant decrease in depressive symptoms [21,23,28,34,36].

- Fatigue

Three meta-analyses from two [21,29] to five [35] studies were conducted to assess the effect of VR-based interventions on fatigue. Only one revealed a significant effect of VR compared to standard care immediately after the intervention [35], but this effect did not persist 48 h later [35]. There was substantial heterogeneity in all pooled data. Individual studies showed mixed results. While some studies reported a beneficial effect [20,23,25,28], others noted small or no effects [33,36].

- Other psychological outcomes

Three meta-analyses from the same two preliminary studies [22,26,31] indicated that VR significantly enhanced outcomes related to fear of medical procedures (e.g., peripheral intravenous catheter placement). However, Czech and colleagues [31] did not pool the data due to high heterogeneity, while others reported low [26] and moderate heterogeneity [22]. In contrast, one study found that fear levels were lower but did not reach statistical significance [20]. Inconclusive findings were observed on distress symptoms: some studies argued that VR was effective [22,23], whereas others did not confirm these findings [35,36]. Only one study focused on the effectiveness of VR on quality of life [22]. However, the pooled results of the two studies did not reveal a statistically significant difference between groups. Finally, VR appeared useful in addressing patients’ comprehension and knowledge of treatment procedures [34], improving the treatment experience [34], and altering time perception [23].

#### 4.2.2. Physical Outcomes

- Shoulder Range of Motion (ROM)

The effect of VR on shoulder ROM and related outcomes (e.g., abduction, flexion, internal rotation) was assessed through three meta-analyses including two [28] to four [25,27] of the same preliminary studies. Despite high levels of heterogeneity in most cases, pooled results indicated that VR was more effective than standard training methods (e.g., routine rehabilitation training, proprioceptive neuromuscular facilitation, scar tissue massage, and passive mobilization) for improving shoulder ROM outcomes.

- Cognitive functions

The effect of VR on cognitive functions such as verbal memory, executive function, and processing speed was investigated by three meta-analyses [25,27,28] and one review [21]. However, it concerned the three same preliminary studies. The heterogeneity of the pooled data ranged from low to high. Significant improvements were found in all but one study [28].

- Other physical symptoms

Few studies revealed a significant positive effect of VR on the incidence of complications after surgery, notably postmastectomy lymphedema [23,25,33]. Similarly, VR showed benefits in reducing fear of movement [25] and improving dynamic postural control [23,33], while not inducing noteworthy cybersickness symptoms [22,23,25], which refers to unpleasant physiological symptoms (e.g., nausea, dizziness), caused by exposure to a virtual environment. More inconsistent results were found for biological outcomes (i.e., heart rate, blood pressure): while some studies reported improvements [23], others failed to draw such clear conclusions [35]. Furthermore, no significant effect was observed on hand grip strength [25,27].

## 5. Discussion

The aim of our umbrella review was to summarize evidence of the current state of research on VR-based interventions in oncology. VR encompasses a broad spectrum of technologies and contents. As mentioned, VR-based interventions can be classified into three main types: distraction, rehabilitation, and education. Our report revealed that the most common use of VR is one-off exposure to distract patients from anticancer treatment and painful procedures, with natural or game-based content. In addition, our descriptive analysis of the effects of VR showed that the most convincing results concerned the reduction in anxiety, pain, and depression. The AMSTAR-2 assessment revealed that the majority of the included reviews had one or more methodological weaknesses. These limitations could potentially impact the reliability of the pooled evidence.

The impact of VR-based interventions on various health-related outcomes, such as fatigue and physical and cognitive functions, has also been assessed. However, the current literature shows less consistent results, and there are not enough data to draw meaningful conclusions. For instance, VR-based interventions showed minimal or no improvement in fatigue. Cancer-related fatigue is a complex and multidimensional symptom, affecting the emotional, physical, and cognitive spheres [38]. The effect of VR on fatigue has been primarily evaluated in patients undergoing chemotherapy infusions [25,29,32,33,35,36]. Future research should assess its impact across a broader spectrum of cancer treatments, including less studied modalities such as immunotherapy or radiotherapy. This expanded approach could provide a more comprehensive understanding of VR’s potential in managing cancer-related fatigue across various treatment contexts. However, current VR interventions may not adequately address the underlying mechanisms of cancer-related fatigue. The often short-term nature of the VR interventions studied in this review may be insufficient to significantly impact a chronic symptom like fatigue. Thus, future research should explore longer-term or specifically designed VR interventions aimed at helping patients regain control over this symptom.

In addition, the heterogeneity of studies has sometimes limited the strength of conclusions. Indeed, a high level of heterogeneity was almost consistently observed across pooled studies, notably regarding methodologies and technologies used. This variability should not be overlooked, as it can lead to erroneous conclusions or to an over-/underestimation of the effects observed. Since the early use of VR in healthcare [39], there have been major advances in publication standards and technologies. Future clinical trials should be aligned with existing recommendations (e.g., CONSORT) and implement standardized protocols to improve the quality and reliability of results [25,29,32]. In addition, only three reviews reported data on cybersickness symptoms induced by the interventions [22,23,25], which have been a recurring issue since the early development of VR technology. These symptoms should be evaluated in a more systematic way to ensure the safety of the interventions implemented.

Only 8 of the 18 reviews focused exclusively on interventions of a single type [24,26,29,30,32,34,35,36] and 1 performed subgroup analysis by intervention type [22]. Due to the varied therapeutic objectives, content, equipment, target populations, and clinical contexts, these interventions cannot be effectively assessed as a group. Only two reviews performed meta-analyses by including preliminary studies evaluating immersive VR-based distraction interventions within a specific population (i.e., pediatric patients) [26,35].

VR-based interventions should be analyzed in a more refined and discriminating way. This approach would help to identify their specific mechanisms of action and allow for content adjustments to maximize their effects. For instance, Comparcini and colleagues [30] found that nonimmersive VR-based distraction had no added benefit over standard care, while immersive VR showed positive effects. In contrast, nonimmersive VR-based rehabilitation showed benefits for physical and cognitive functions [25]. This suggests that immersion may not be a necessary condition for effective rehabilitation. Pooling data from different interventions complicates the identification of the key components responsible for the effects observed. Current studies tend to focus on assessing the overall effects of VR interventions, often overlooking potential interindividual differences. However, certain patient profiles may be more receptive to interventions than others [40]. To gain a deeper understanding of the underlying mechanisms in VR, it is crucial to take into account relevant individual variables in a medical context, such as self-efficacy, engagement, locus of control, or coping strategies.

Moreover, heterogeneity exists within the interventions themselves. For example, VR-based distraction includes a wide range of content, from passive exposure to beaches, lakes, and mountains to more active games. While games can stimulate engagement, entertainment, and enjoyment [41], natural environments may be more effective at reducing stress and inducing a state of relaxation [42]. Several theories explain links between nature and emotional well-being (e.g., The Biophilia Hypothesis, Stress Reduction Theory, Attention Restoration Theory) [43]. Green and blue environments have shown particularly notable results [44]. When designing future interventions, it is essential to draw on existing theories, as current studies often lack a solid theoretical foundation. In addition, there is a need for theoretical models that go beyond the attentional mechanism [45]. This theoretical grounding will enhance our understanding of how VR affects patients and enable the development of interventions that are more tailored to patient needs and better targeted toward therapeutic outcomes.

Several researchers agree that advancing VR-based interventions in cancer care requires a more multifaceted approach [20,22,24,32]. They suggest that simply exposing patients to visually pleasant content may not be enough and that integrating therapeutic factors (e.g., relaxation, mindfulness, meditation) could provide more beneficial long-term effects [22,24,32]. Additionally, the available literature highlights various factors that could enhance user experience and well-being, including familiarity and environmental preference, the integration of natural elements (e.g., sounds of nature and animals), immersion levels, and the sense of presence [46,47]. The use of complementary tools, such as artificial intelligence, physiological indexes, and electroencephalograms, may also optimize the effects of VR [21,22]. In addition, current VR-based education focuses primarily on the transmission of procedural information about treatment. Yet, sensory information seems to be more effective in reducing patient anxiety [48]. These insights should guide the design of future interventions.

According to the systemic approach in psycho-oncology, cancer care should be conceptualized as an integrated system involving patients, family caregivers, and healthcare professionals [49]. This holistic approach recognizes the interdependence of patient outcomes, caregiver well-being, and healthcare quality. In this context, VR could play a crucial role in supporting family caregivers, thereby strengthening the overall support provided to patients. As highlighted by a recent meta-analysis [50], family caregivers encounter various emotional challenges, including uncertainty and feelings of unpreparedness, which have a negative impact on their well-being and functionality. In line with these findings, VR-based distraction and education could also benefit caregivers by facilitating clearer communication, improving access to information, and providing valuable psychological support. These interventions may alleviate the demands on healthcare professionals who sometimes lack the time to respond efficiently to the needs of patients and family caregivers.

Our report underscores the need to redefine the notion of VR, particularly in light of its ongoing evolution. The prevailing definition considers VR as a technology that depicts a computer-generated 3D environment, accessible via various visual presentation devices. However, the emergence of new forms of content such as 360° video, and the increased accessibility to HMDs (e.g., technological advances and lower costs), challenge this conventional understanding. For instance, some researchers use “active video games” [34] to describe games based on platforms such as Xbox 360 Kinect or Nintendo Wii, while others refer to them as VR-based interventions, e.g., [17,35]. This terminological ambiguity highlights the importance of establishing clear criteria for classifying VR and distinguishing it from other forms of interactive digital intervention. This clarification is essential to avoid ambiguity in scientific communication and to ensure consistency in research. It is also worth noting that most of the reviews analyzed included preliminary studies from 20 years ago. Technological advances in the quality of graphics and increased realism of the content presented to patients may have impacted the results, thus potentially explaining some of the inconsistencies in the conclusions reported.

To our knowledge, this is the first umbrella review to synthesize evidence from systematic reviews and meta-analyses on VR-based interventions in cancer patients. This report can serve as a foundation for future research by highlighting the varied nature of VR interventions, their variable effects, and the methodological challenges that arise when evaluating them.

## 6. Limitation

Our review has some limitations. First, it is possible that some relevant reviews have been excluded due to specific selection criteria. Our focus on cancer-related interventions only, the inclusion of English- and French-language reviews, the exclusive examination of systematic reviews, and the use of four databases may have limited the scope of our findings. This may have limited the representativeness of our umbrella review. Additionally, as mentioned above, terminological confusion surrounding VR may have affected our keyword search results. Finally, the included reviews cover diverse populations, both in terms of diagnosis and treatments received, which may limit the ability to extrapolate our results to specific groups of patients or treatments.

## 7. Conclusions

The overall findings of our report indicate that VR is an effective adjuvant intervention across various medical settings, encouraging its integration throughout the patient care pathway. However, the findings also emphasize the need for a more nuanced understanding of its applications. To date, VR-based interventions are insufficiently documented and protocolized. Future research should focus on deepening our understanding of relevant content to optimize therapeutic effects. This requires expanding our knowledge of the mechanisms of action behind each intervention and designing them on a solid theoretical basis. Content must be tailored and individualized to ensure consistency and effectiveness, acknowledging the heterogeneous nature of cancer diagnoses (i.e., populations, treatments, and procedures involved). Additionally, there is a need for more robust trials and standardized content to ensure proper replication and implementation in everyday practice.

## Figures and Tables

**Table 1:** Study characteristics.

Author(s) (Year)	Country	Meta-Analysis	Objective(s)	Population	No. and Design of Studies Included (No. of Participants)	Comparator(s)	Outcome(s)
Chow et al. (2021) [24]	Australia	No	To provide “context and recommendations for improving the robustness of future studies with cancer patients”	Adult and pediatric with various types of cancer (e.g., leukemia, breast, lung)	9: 4 RCTs and 5 cross-over design RCTs (401)	Standard care (e.g., conscious sedation, local anesthetic, antiemetic medication) Note: standard care sometimes included the possibility of reading a book, watching television, and listening to television, which can be considered as forms of distraction	Pain, anxiety
Bu et al. (2022) [25]	China	Yes	“To qualitatively and quantitatively examine the effects of VR-based interventions on breast cancer survivors”	Adult with breast cancer	12: 4 RCTs, 2 cross-over design RCTs, 3 pre–post-test designs, 2 quasi-experimental designs, and 1externally controlled trial (605)	Standard care (e.g., painkiller only, standard education, standard physical training), proprioceptive neuromuscular facilitation, music therapy, standardized physical therapy	Shoulder range of motion, hand grip strength, anxiety, depression, pain, cognitive function (i.e., verbal memory), fatigue, incidence of complications, cybersickness symptoms, fear of movement
Cheng et al. (2022) [26]	China	Yes	“To evaluate the effect of VR technology on relieving pain and anxiety related to children with cancer is needed in order to provide scientific evidence-based basis for relieving pain and anxiety in clinical nursing practice”	Pediatric (<18 years) diagnosed with any type of cancer	6 RCTs (379)	Standard care, video (same as VR condition) via iPad	Pain, anxiety, fear
Tian et al. (2023) [27]	China	Yes	“To examine the effects of VR-based interventions on breast cancer-related symptom management of anxiety, depression, pain, cognitive function, grip, shoulder range of motion (ROM), or upper extremity function”	Adult with breast cancer	14: 6 RCTs, 6 quasi-RCTs, and 2 cross-over design RCTs (797)	Standard care or procedure (e.g., usual rehabilitation training, pretreatment teaching, oral or intravenous morphine), proprioceptive neuromuscular facilitation, rehabilitation exercise, music therapy	Anxiety, depression, pain, cognitive function, upper extremity function, hand grip strength, shoulder range of motion
Wu et al. (2023) [22]	China	Yes	“To pool high-quality data from RCTs to systematically analyze and evaluate comprehensive evidence on the effects of VR-based interventions on anxiety, pain, depression, distress, fear and quality of life in patients with cancer”	Adult and pediatric with various types of cancer (e.g., breast, colorectal, prostate)	12 RCTs (825)	NS	Anxiety, pain, depression, fear, distress, quality of life
Zeng et al. (2019) [28]	China	Yes	“To report on the most recent studies using VR-based interventions for symptom management in patients with cancer, and to quantitatively evaluate the efficacy of VR-based intervention in cancer-related symptom management”	Adult with various types of cancer (e.g., metastatic, breast, lung, hematologic malignancies)	6: 1 RCT, 1 case-controlled trial, and 4 pre–post-test designs with a single arm (225)	Standard care	Anxiety, depression, fatigue, pain, cognitive function (i.e., verbal memory, processing speed)
Zhang et al. (2022) [21]	China	Yes	“To determine the effectiveness of VR-based intervention on the symptoms and rehabilitation management in patients with breast cancer”	Adult with breast cancer	8: 6 RCTs and 2 quasi-experimental designs (NS)	Standard care (e.g., traditional rehabilitation training, regular health education, standard upper limb physical therapy treatment, painkiller), music therapy	Anxiety, fatigue, abduction
Yazdipour et al. (2023) [23]	Iran	No	“To look into the benefits and drawbacks of VR interventions for breast cancer patients”	Adult with breast cancer	18: 5 pre–post-test designs, 5 cross-over designs, 4 RCTs, 1 cross-sectional design, 1 experimental design, 1 quasi-experimental design, and 1 quasi-RCT (842)	NS	Any outcome related to mental or physical sphere (e.g., anxiety, pain, fatigue, strength, function metrics)
Burrai et al. (2023) [29]	Italy	Yes	“To assess the effectiveness of VR on anxiety, fatigue and pain in patients with cancer during chemotherapy, and to provide evidence for decision-making in clinical nursing practice”	Adult with various types of cancer (e.g., breast, lung, colon)	8: 4 RTCs and 4 cross-over designs (453 and 15 dropouts were reported)	Standard care (e.g., painkiller only), integrative therapies (i.e., guided imagery, green therapy, music therapy)	Anxiety, fatigue, pain
Comparcini et al. (2023) [30]	Italy	No	“To assess the effectiveness of VR for the management of pain and/or anxiety in children and adolescents with hematological or solid cancer”	Pediatric (<18 years) with hematological or solid cancer	13: 4 RCTs, 3 quasi-experimental designs, 2 pilot RCTs, 1 cross-over design RCT, 1 parallel group design, 1 cross-over design, and 1 interrupted time series study (644)	Standard care (e.g., no distraction, parental presence, topical anesthetic only), video (same as VR condition) via iPad	Pain, anxiety
Sansoni et al. (2022) [20]	Italy	No	“To explore the state of the art of psychological and educational interventions among adult and pediatric cancer patients”	Adult and pediatric with various types of cancer (e.g., cervical, breast, colon)	20: 10 RCTs, 4 cross-over designs, 2 within-subjects designs, 1 observational study, 1 prospective single-arm study, 1 externally controlled trial, and 1 interrupted time series study (NS)	Standard care (e.g., waiting list, parental presence, conscious sedation, painkiller only, face-to-face education), video (same as VR condition) via iPad, green therapy room	Any outcome related to psychological sphere (e.g., anxiety, depression, pain, well-being)
Ahmad et al. (2020) [9]	Jordan	No	“To summarize and evaluate the methodological quality of primary clinical trials on VR for management of pain and anxiety among patients with cancer and to analyze the effectiveness of VR in the reviewed studies”	Adult and pediatric with various types of cancer (e.g., breast, lung, colorectal, leukemia, solid tumor)	13: 5 RCTs and 8 quasi-experimental designs (522)	Standard care (e.g., conscious sedation, painkiller, pretreatment teaching, antiemetic medications), distraction without VR (i.e., playing with the virtual program displayed on the computer screen)	Pain, anxiety
Czech et al. (2023) [31]	Poland	Yes	“To investigate the effects of VR and AVG on fear, physical functions, and quality of life”	Pediatric (<18 years) with various type of cancer (e.g., leukemia, lymphoma)	9: 6 RCTs and 3 cross-over design RCTs (393, not specified for 1 study)	Standard care (e.g., waiting list, anesthesia spray), video (same as VR condition) via iPad	Pain, anxiety, fear
Rutkowski et al. (2021) [32]	Poland	Yes	“To analyze the effectiveness of VR intervention on anxiety and fatigue in cancer patients undergoing chemotherapy”	Adult with various types of cancer (e.g., breast, colon)	6: 3 RCTs and 3 cross-over designs (506)	Standard care (e.g., painkillers only, no more details) Note: in one study, patients in control group could choose any activity (e.g., watching television, reading)	Anxiety, fatigue
Zasadzka et al. (2021) [33]	Poland	No	“To conduct an overview of the clinical studies where researchers used VR intervention in breast cancer patients as a tool in cancer rehabilitation of this patients’ group”	Adult with breast cancer (3 studies included other types of cancers such as gynecological, colon, or lung cancer)	11: 2 RCTs, 4 cross-over designs, 2 quasi-experimental designs, 1 pre–post-test design, and 1 pilot study with a single arm (619)	Standard care (e.g., proprioceptive neuromuscular facilitation, no distraction, standardized physical therapy)	Physical functions (primary), mental sphere and pain (secondary)
Grilo et al. (2023) [34]	Portugal	No	“To understand the impact of radiotherapy educational sessions with VR on oncologic adult patients’ psychological and cognitive outcomes related to the treatment experience”	Adult with various types of cancer (e.g., breast, lung, prostate)	8: 6 pre–post-test designs with a single arm and 2 quasi-experimental designs (376)	Standard education (e.g., written and/or oral explanations)	Knowledge, anxiety, other psychological variables related to the RT experience
Gautama et al. (2023) [35]	Taiwan	Yes	“To investigate the effects of IVR on psychological aspects (anxiety, distress, depression, and distress), physical aspects (fatigue and pain), and objective measurements (blood pressure and heart rate) in cancer patients receiving chemotherapy”	Adult and pediatric with various types of cancer (e.g., breast, ovarian, lung, colon)	12 RCTs (804)	Relaxing music, standard care (i.e., doing nothing)	Anxiety, depression, distress, fatigue, pain, systolic blood pressure, heart rate
Leggiero et al. (2020) [36]	USA	No	“To explore the existing literature for VR-related interventions for symptom management in adult PBT and other solid-tumor patients, which will guide future development of VR interventions in these populations”	Adult and pediatric with various types of cancer (e.g., breast, lung, leukemia)	13: 3 RCTs, 3 cross-over designs, 3 pre–post-test designs, 3 feasibility studies, and 1 secondary data analysis combining results from three previous experimental trials (721)	NS	Pain, anxiety, distress, depression, mood, fatigue, fear

Note: RCT, randomized controlled trial; NS, not specified.

**Table 2:** Methodological quality of the included reviews.

Author(s) (Year)	AMSTAR-2 Items															
	1	2 *	3	4 *	5	6	7 *	8	9 *	10	11 *	12	13 *	14	15 *	16
Ahmad et al. (2020) [9]	Y	N	Y	P	Y	Y	Y	Y	P	N	N/A	N/A	Y	Y	N/A	N
Bu et al. (2022) [25]	Y	Y	Y	P	Y	Y	N	P	P	N	N	N	Y	N	N	Y
Burrai et al. (2023) [29]	Y	Y	N	P	Y	Y	N	P	Y	N	Y	Y	Y	Y	Y	Y
Cheng et al. (2022) [26]	Y	N	Y	P	Y	Y	N	P	Y	N	Y	N	N	Y	N	Y
Chow et al. (2021) [24]	Y	P	Y	P	Y	Y	Y	P	P	N	N/A	N/A	Y	N	N/A	Y
Comparcini et al. (2023) [30]	Y	N	Y	P	Y	Y	Y	Y	Y	N	N/A	N/A	N	N	N/A	Y
Czech et al. (2023) [31]	Y	P	N	P	Y	Y	Y	Y	Y	N	Y	Y	N	N	N	Y
Gautama et al. (2023) [35]	Y	P	N	P	Y	Y	N	Y	Y	N	Y	Y	N	Y	Y	N
Grilo et al. (2023) [34]	Y	N	N	P	Y	Y	N	Y	P	N	N/A	N/A	N	N	N/A	Y
Leggiero et al. (2020) [36]	Y	N	Y	P	Y	Y	N	Y	N	N	N/A	N/A	N	Y	N/A	Y
Rutkowski et al. (2021) [32]	Y	N	Y	P	Y	Y	Y	P	Y	N	Y	N	N	N	N	Y
Sansoni et al. (2022) [20]	Y	N	Y	P	Y	Y	N	P	Y	N	N/A	N/A	N	N	N/A	N
Tian et al. (2023) [27]	Y	N	Y	P	Y	Y	Y	P	P	N	Y	N	N	N	N	Y
Wu et al. (2023) [22]	Y	N	Y	P	Y	Y	N	P	Y	N	Y	N	N	N	N	Y
Yazdipour et al. (2023) [23]	Y	N	Y	P	Y	Y	N	P	Y	N	N/A	N/A	N	N	N/A	Y
Zasadzka et al. (2021) [33]	Y	N	Y	P	Y	Y	N	P	Y	N	N/A	N/A	N	N	N/A	Y
Zeng et al. (2019) [28]	Y	N	Y	P	Y	Y	N	P	P	N	N	N	N	Y	N	Y
Zhang et al. (2022) [21]	Y	N	Y	P	Y	Y	N	P	Y	N	Y	N	Y	Y	N	Y

Note: N, no; Y, yes; P, partly; N/A, not applicable since no meta-analysis was conducted. * Critical domains.

## Data Availability

No new data were created or analyzed in this study. Data sharing is not applicable to this article.

## Supplementary Materials

The following supporting information can be downloaded at: https://www.mdpi.com/article/10.3390/cancers16233943/s1, PRISMA 2020 Checklist; ASMTAR-2 Items.
